# Twitter use in scientific communication revealed by visualization of information spreading by influencers within half a year after the Fukushima Daiichi nuclear power plant accident

**DOI:** 10.1371/journal.pone.0203594

**Published:** 2018-09-07

**Authors:** Masaharu Tsubokura, Yosuke Onoue, Hiroyuki A. Torii, Saori Suda, Kohei Mori, Yoshitaka Nishikawa, Akihiko Ozaki, Kazuko Uno

**Affiliations:** 1 Department of Radiation Protection, Soma Central Hospital, Soma, Fukushima, Japan; 2 Science for Innovation Policy Unit, Center for the Promotion of Interdisciplinary Education and Research, Kyoto University, Kyoto, Japan; 3 School of Science, The University of Tokyo, Tokyo, Japan; 4 Department of Physics and Astronomy, Graduate School of Science, Kyoto University, Kyoto, Japan; 5 Hamamatsu University School of Medicine, Hamamatsu, Aichi, Japan; 6 Department of Health Informatics, Graduate School of Medicine and Public Health, Kyoto University, Kyoto, Japan; 7 Department of Surgery, Minami-soma Municipal General Hospital, Minami-soma, Fukushima, Japan; 8 Louis Pasteur Center for Medical Research, Kyoto, Japan; University of Padua, ITALY

## Abstract

Scientific communication through social media, particularly Twitter has been gaining importance in recent years. As such, it is critical to understand how information is transmitted and dispersed through outlets such as Twitter, particularly in emergency situations where there is an urgent need to relay scientific information. The purpose of this study is to examine how original tweets and retweets on Twitter were used to diffuse radiation related information after the Fukushima Daiichi nuclear power plant accident. Out of the Twitter database, we purchased all tweets (including replies) and retweets related to Fukushima Daiichi nuclear power plant accident and or radiation sent from March 2^nd^, 2011 to September 15^th^, 2011. This time frame represents the first six months after the East Japan earthquake, which occurred on March 11^th^, 2011. Using the obtained data, we examined the number of tweets and retweets and found that only a small number of Twitter users were the source of the original posts that were retweeted during the study period. We have termed these specific accounts as “influencers”. We identified the top 100 influencers and classified the contents of their tweets into 3 groups by analyzing the document vectors of the text. Then, we examined the number of retweets for each of the 3 groups of influencers, and created a retweet network diagram to assess how the contents of their tweets were being spread. The keyword “radiation” was mentioned in over 24 million tweets and retweets during the study period. Retweets accounted for roughly half (49.7%) of this number, and the top 2% of Twitter accounts defined as “influencers” were the source of the original posts that accounted for 80.3% of the total retweets. The majority of the top 100 influencers had individual Twitter accounts bearing real names. While retweets were intensively diffused within a fixed population, especially within the same groups with similar document vectors, a group of influencers accounted for the majority of retweets one month after the disaster, and the share of each group did not change even after proven scientific information became more available.

## Introduction

The term scientific communication is defined as communicating scientific information to non-experts in the general public [1]. In this age of information overload, appropriate scientific communication is critical to raise the scientific literacy of the public, to implement policies based on evidence, and to improve the well-being of citizens [2].

It was common in the past to provide the general public with one-directional information through classical mass media outlets such as newspapers, televisions, and radios [3]. Recently however, social media platforms, such as Twitter and Facebook, have been playing increasingly important roles as media through which to disseminate and receive scientific information [4, 5]. In fact, it is estimated that approximately 60% of the general public rely on social media as a source for scientific information [6]. Social media platforms enable real-time communication with rapid propagation over a wide demography [7]. In regard to scientific communication however, there are several drawbacks to using social media. In particular, there is concern about the spread of scientifically inappropriate or inaccurate information through erroneous rumors or hoaxes during times of natural or other disasters [8, 9]. For example, there were prior incidences of inappropriate scientific information regarding vaccine efficacy and cancer treatments being disseminated through social media [10]. Therefore, to use social media effectively for scientific communication, it is important to identify how to make good use of the properties of these media in the future.

Twitter is a social media platform where registered users can create posts containing up to 280 characters and attach images. At the time of this study however, the limit in place was 140 characters, and even now Japanese tweets fall outside the scope of this deregulation. Twitter users can follow each other freely and spread information more broadly compared to Facebook. [11]. On Twitter, the relationship between users who are followers and those who are being followed forms a social network, and retweeting or replying to another user’s tweet is the way to distribute and propagate information. Retweeting is the act of spreading information to one’s followers by quoting verbatim the tweet of other users [12]. In this way Twitter can be used to diffuse scientific information, and its role has been increasing in recent years due to its high real-time capabilities and ease of exchanging information with other users.

The advantage of Twitter is that it allows direct communication between people who are too far away socially as well as physically in everyday life. Especially, at the time of a social phenomenon, such as a disaster that attracts public attention, related tweets rapidly increase [13]. As such, Twitter is regarded as a very useful social media tool to obtain necessary information, spread information, and ask for help in case of a disaster [14, 15]. Despite Twitter being a platform that plays a key in the exchange of current information, there are limited reports that focus on how scientific information diffuses, and how Twitter is useful for scientific communication within the first few months after a disaster.

The Great East Japan Earthquake and the subsequent Fukushima Daiichi nuclear accident, which occurred on March 11^th^, 2011, resulted in radioactive contamination and radiation exposure to the public [16]. Residents in the surrounding areas had long-term radiation exposure and the fear of the contamination spreading resulted in social unrest across Japan [17]. In response to this situation, one-directional and conventional scientific communication regarding radiation was released from various government and private sources [18]. However, in addition to a wide range of perceived problems (such as health, societal, and lifestyle) caused by radiation pollution, there were some stakeholders making scientifically erroneous assertions particularly about low-dose radiation and its health effects. Many conflicting opinions circulated and caused confusion among the general public [19]. As a result, residents were at a loss as to whom to believe, and the public’s trust in science itself was lost [20].

Social media, in particular Twitter, was actively used for both direct communication and for transmission and exchange of scientific information at the time of the earthquake [21–24], especially in the affected areas. However, many reports on the subject have only described the phase immediately after the Fukushima nuclear power plant accident including evacuation and logistics [25, 26], and there is insufficient information on how Twitter was used for scientific communication of radiation-related issues. Assessing how Twitter was used after the radiation accident is very useful in order to clarify how social media is used in the world of scientific communication.

In the present study, we used Twitter data up to six months after the accident, to examine how Twitter was used, especially as it relates to retweets, and to see the spreading of scientific information on radiation. This study will provide useful information for scientists to understand the background of distrust in science after the Fukushima Daiichi nuclear power plant accident, and to better understand the method of appropriate scientific information dissemination on social media platforms, such as Twitter should there be a future crisis.

## Materials and methods

### Tweet and retweet data used

Tweets and retweets used in this research was purchased from NTT DATA Corporation. NTT DATA, an IT company, is a member of NTT (Nippon Telegraph and Telephone Corporation) Group, the largest telecommunications company in Japan. NTT DATA is Twitter’s intermediary in Japan authorized to give customers paid access to tweets. For this research, we gave NTT DATA a list of Japanese words, phrases and expressions related to radiation and radioactivity resulting from the Fukushima Daiichi nuclear power plant (Table 1). Using this list of keywords, NTT DATA extracted and compiled the contents of tweets and retweets (including replies) written in Japanese that were sent on between March 2^nd^, 2011 to September 15^th^, 2011 (i.e. the first six months after the Great East Japan Earthquake). This data was purchased and used in our analysis.

**Table 1:** Words used to select tweets regarding radiation and Fukushima disaster. (Japanese and corresponding English translation).

放射	radio- / radia- (which includes radiation, radioactive, radioactivity etc.)
被ばく, 被曝, 被爆	exposure
除染	decontamination
線量	dose
ヨウ素	iodine
セシウム	cesium
Sv, mSV, μSV, uSV, msv, μsv, usv, シーベルト	Sv, sievert
ベクレル	becquerel
Bq	Bq
ガンマ線, γ線	gamma ray, γ‐ray
核種	isotope
甲状腺, 甲状線	thyroid
チェルノブイリ	Chernobyl
規制値	regulation value
基準値	standard value
学会	academic society
警戒区域	no-entry zone
避難区域	evacuation zone
産科婦人科	obstetrics and gynecology
周産期・新生児医	perinatal and neonatal care
日本疫	Japanese society of epidemiology
核医	nuclear medicine
電力中央	central electric
学術会議	science council
環境疫	environmental epidemiology
物理学会	Physical Society
プルトニウム	plutonium
ストロンチウム	strontium
暫定基準	provisional standard
暫定規制	provisional regulation
屋内退避	sheltering
金町浄水場	Kanamachi Water Purification Plant
出荷制限	shipment restriction
管理区域	control area
避難地域	evacuation area
モニタリング	monitoring
スクリーニング	screening
ホットスポット	hot spot
汚染	contamination
検査 AND (食品 OR 水 OR 土)	inspection AND (food OR water OR soil)
リスク AND (がん OR ガン OR 癌)	risk AND cancer
影響 AND (妊婦 OR 妊娠 OR 出産 OR 子ども OR 子供 OR こども OR 児)	effect AND (pregnant woman OR pregnancy OR childbirth OR child)
母子避難	mother and child evacuation
避難弱者	people having difficulty in evacuation
自主避難	voluntary evacuation
避難関連死, 避難死	death associated with evacuation
(福島 OR ふくしま OR フクシマ) AND (避難 OR 米 OR 野菜 OR 牛肉 OR 食品 OR 産 OR 安全 OR 安心 OR 不安 OR 検査)	Fukushima AND (evacuation OR rice OR vegetable OR beef OR food OR product OR safety OR relief OR anxiety OR inspection)

All tweets extracted by NTT DATA that had at least one of the keywords and key phrases shown in Table 1 were included in the analysis. Keywords and phrases were chosen to analyze events and facts related to radiation and not the effects of the earthquake and tsunami; they also did not include emotional words that expressed fear, anxiety or anger about the radiation. All members of our research team agreed that the search terms shown in Table 1, was a highly accurate representation of the scope of the Fukushima nuclear power plant accident and radiation. Due to our allowed fiscal budget, this research was limited to 50 million tweets, as a result "nuclear power plant" and "Fukushima" and other terms that are related, but not critical to our research, were not used as independent search terms.

The number of tweets and retweets transmitted during the study period, and the transition of the retweet ratio among all tweets were examined. Retweets were counted by the number of accounts that posted the same tweet; for example, if 10 different accounts each retweeted one tweet posted by user-A, the number of retweets was counted as ten.

### Definition of “influencers” and classification of their tweet contents

We found that the majority of the retweets were based on original posts sent out by a few hundred accounts. The top 2% of accounts were the source of original posts that received 80.3% of all retweets during the study period. Thus, the top 100 accounts that were retweeted frequently were identified and defined as “influencers”.

To classify the contents of their tweets, we calculated tweet's document vectors for each influencer’s account. The method is as follows: using the text that appeared in all the tweets used in this study and the article text of Japanese Wikipedia as corpus, each Japanese document was separated with spaces using Japanese morphological analysis engine MeCab [27]. For the dictionary, ipadic and mecab-ipadic-neologd were used [28]. In this way we derived 390,681,577 words from 12,219,497 tweets, and 397,785,864 words from 1,072,888 Wikipedia articles. The number of unique words combined was 171,644. Gensim version 2.3, a natural language processing library for Python programming was used to execute Doc2Vec [29]. The default parameter setting of genism was used for learning with 100 dimensions of the output vector. Python code can be found in the following URL (https://github.com/likr/twitter-analysis2018/tree/master/scripts).

*K*-means method was applied to the document vector to classify each influencer [30]. Of the top 100 influencer accounts examined during the study period, 99 accounts were still active as of June 2017. Five accounts of outliers that did not constitute the same clusters with other influencer accounts have been removed, so 94 accounts were used for the final clustering.

First, five clusters were identified in the *k*-means method based on the Elbow method [31]. These five clusters were then grouped according to the contents of their tweets regarding radiation and whether the clusters included media accounts or not. Finally, we classified the influencers into three groups, and examined the number and the ratio of retweets over the study period.

### Visualization of radiation information spreading by influencers

In order to visualize the spread of radiation information by influencers, we built a retweet network centered on influencers. A retweet network is a weighted directed graph linking the relationship that account *A* has retweeted influencer *X* for *n* number of times. We visualized the center of the retweet network using only the top 20 influencers and accounts with more than 5 retweets. The Fast Multipole Multilevel Method [32] (FM3) implemented in the Open Graph Drawing Framework [33] was used to set the coordinate positions of nodes.

### Protection of personal information accompanying tweet and account data use

The data of this study was received from Twitter, Inc. and are in accordance with the company's user agreement for the handling of personal information. Due to contractual agreements with NTT DATA, the purchased data used in this study cannot be shared. However, researchers can purchase this data through Twitter Inc., or its local intermediary, by specifying the same keywords and key phrases listed in Table 1. The extracted tweets and retweets will then be similar to the ones we purchased from NTT DATA. The Twitter accounts which were used in this study can be accessed individually by the public for free at www.twitter.com. All tweets and retweets generated from these accounts can also be viewed, although they will not be limited to those bearing our keywords listed in Table 1.

## Results

### Overall trends of tweet and retweet

**Fig 1:**
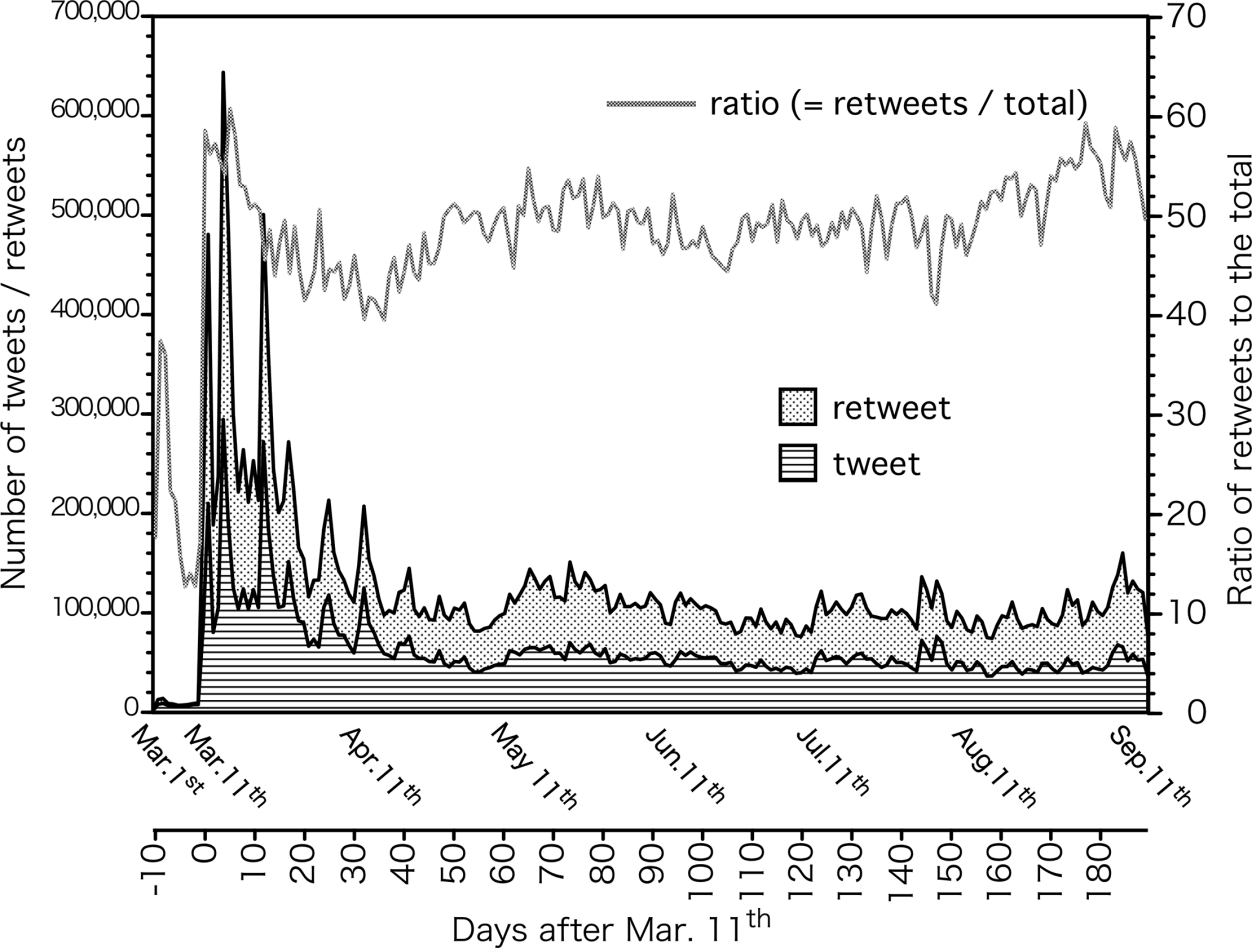
The number of daily tweets and retweets (see the left ordinate) and the ratio of retweets to the total (right ordinate) from March 2^nd^, 2011 to September 15^th^, 2011. The number of retweets is shown on top of the number of tweets.

The total number of tweets and retweets that included the keywords listed in Table 1 during the period from March 2^nd^ to September 15^th^, 2011 was 24,287,299. The number of accounts that sent out tweets or retweets at least once was 1,397,941. Since Japanese is written without space between words, if the Japanese words in Table 1 were a part of a sentence or ID, it was included in the study. On the other hand, in the case of an English word like Sv, it was not used as a search term unless it exists as an independent English word of Sv. When radio- is searched in Japanese, compound words such as heat radiation, emissivity, radiologist, etc. are included in the search result. Regarding contamination, it has related words such as environmental pollution and nuclear pollution. In order to clarify the extent of how such unrelated tweets are included in the present study, we randomly extracted 1000 tweets, and confirmed that there are 116 tweets which seem to be irrelevant to the present research topic. Using that as an estimate, we predict that unrelated tweets accounted for 11.6±2.6% of total tweets assessed in this study. The number of tweets per account fell between 0 to 61,037 with a median of 1; the number of retweets sent out per account was 0 to 36,716 with a median of 1. Fig 1 shows the number of tweets and retweets per day during the study period.

The number of tweets and retweets per day that included the keywords shown in Table 1 increased sharply after March 11^th^, 2011 (= day 0 of the Great East Japan Earthquake). The number remained high in the first month, but it decreased drastically after the second month (around 100,000 cases per day). The maximum was 643,603 on March 15^th^, 2011 (= day 4), the minimum was 74,274 on August 15^th^, 2011 (= day 157). The average number of tweets and retweets per day for each month after the disaster was 241,529, 109,197, 120,720, 93,854, 103,953, and 99,464 (from 11^th^ March to 10^th^ April, from 11^th^ April to 10^th^ May, from 11^th^ May to 10^th^ June, from 11^th^ June to 10^th^ July, from 11^th^ July to 10^th^ August, and from 11^th^ August to 10^th^ September, respectively). Several spikes were observed in the first month; 12^th^ March (= day 1), 15^th^ March (= day 4), 16^th^ March (= day 5), and 23^rd^ March (= day 12) measured the number of 400,000 or more (480, 573, 643,603, 488,555, and 500,575, respectively). After 1 month (11^th^ April), only 12^th^ April (= day 32) exceeded 200,000 (207,293 tweets and retweets).

During the study period, the total number of retweets was 12.07 million which accounted for 49.7% of all tweets and retweets combined. This retweet ratio remained at around 50% during the study period, but more precisely, it started with a downward trend one month after the disaster and then increased slightly afterwards. The average during the first week of the disaster was 57.3%. The weekly average one month after the earthquake (average for 7 days from 11^th^ April) was 41.4%, and the monthly average during August was 50.3%.

**Fig 2:**
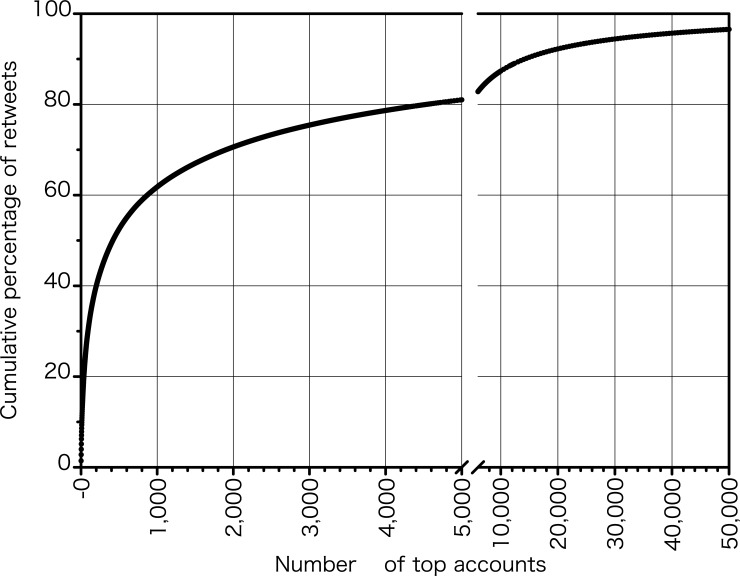
Cumulative percentage of retweets received by top *N* accounts after arranging accounts in descending order of retweet counts.

Fig 2 shows the cumulative percentage of retweets for accounts arranged in descending order of retweet volume. While the number of accounts that received retweet at least once was 232,607, the top 100 influencers accounted for 3.76 million retweets (31.1%) out of 12.07 million retweets. The top 200 accounts received 4.8 million retweets (40.0%), and the top 2% accounts received 80.3% of all retweets.

### Classification of influencers by Doc2Vec

The document vectors of tweets by influencers were calculated using Doc2Vec. Table 2 shows the result of clustering influencer accounts into 5 groups by *k*-means method.

**Table 2:** Classification of influencers by k-means methods.

Cluster		Final Grouping	Individual accounts with real name	Individual accounts with anonymous name	News agency	Other	Deleted accounts	Total
1	A		10	3	0	1	0	14
2	B		21	17	1	0	1	40
3	B		21	4	1	0	1	27
4	C		0	0	5	0	0	5
5	C		1	0	7	0	0	8

Among the 94 influencers that were analyzed, 53 (56%) accounts had real names, 24 (26%) were anonymous, and 14 (15%) were accounts for press-related companies.

In Cluster 1, ten out of 13 individual accounts had real names. Of these ten accounts, four belonged to academia, two were journalists, and one bureaucrat. In Cluster 1, tweets tended to rationally describe the effect of radiation based on facts, and this cluster was defined as group A. A typical tweet is as follows:

“*In 1974 China conducted atmospheric nuclear tests, and radioactive materials fell in Tokyo with rain. I was a student then, and measured people’s hair and clothes with a Geiger counter. The measured values were comparable or larger than those experienced at hospitals in Fukushima. No health problem due to radiation exposure has been reported up to the present for the citizens exposed then in Tokyo*.”

Cluster 2 had 21 out of 38 individual accounts with real names. Two accounts belong to academia, four businessmen, three journalists, and three politicians. In Cluster 3, 21 of the 25 individual accounts had real names. Among them, three accounts belonged to academia, with five journalists and six politicians.

Both Cluster 2 and cluster 3 had many emotional tweets, and criticisms against the government and Tokyo Electric Power Company (TEPCO). Since these two clusters had similar tweet contents regarding radiation, we combined them to create group B. The contents of tweets concerning radiation among group B differed from those among group A. A typical tweet is below:

“*I will repeat it many times! To buy and eat radioactively polluted agricultural and fishery products is showing "support for TEPCO" rather than "assistance for victims"! Why should consumers, at the expense of their own health, help with the damage that should be compensated for by TEPCO? Stop doing this stupid thing now! Do you want to save TEPCO until your children develop thyroid cancer?*”

Cluster 5 consisted of 7 news agencies and one individual account of a journalist. Since clusters 4 and 5 were accounts related to mass media, these two clusters were collectively shown as group C for subsequent counting.

### Trends of retweets in the three influencer groups

**Fig 3:**
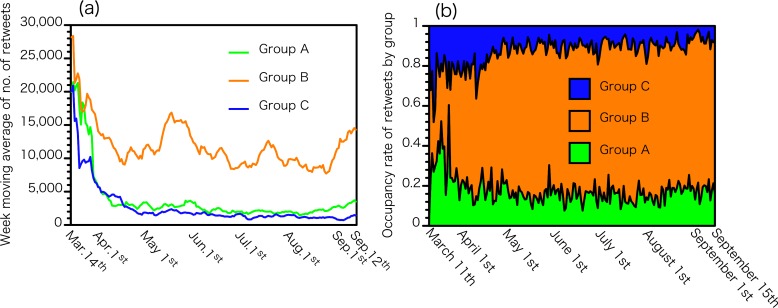
(a) The number and (b) the proportion of retweets that each influencer group accounted for out of the total retweets from March 2^nd^, 2011 to September 15^th^, 2011.

Fig 3 shows (a) the number and (b) the proportion of retweets that each influencer group accounted for out of the total retweets. At the beginning of the disaster, the number of retweets received by group A influencers was almost equal to that of group B, but after one month group B received the majority of retweets, and the situation remained unchanged afterwards. Tweets posted by group C received the lowest number of retweets. Specific bumps were observed in group B in the middle of May and in the middle of July.

### Result of visualizing network diagram

**Fig 4:**
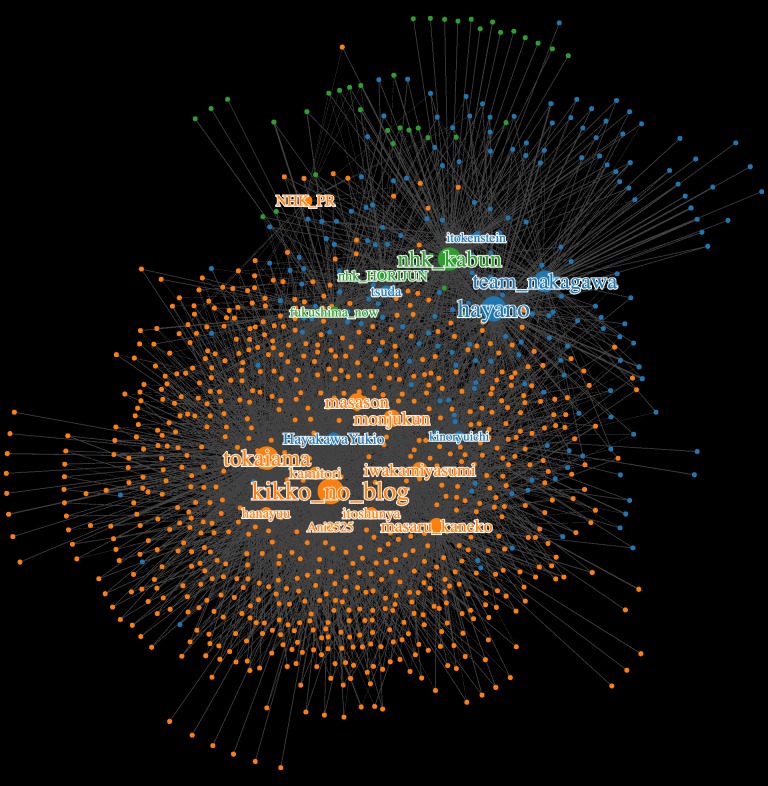
Retweet network diagram of radiation information spreading by influencers.

Fig 4 shows the retweet network diagram of radiation information generated by influencers. A node with an account name as a label represents an influencer. The size and color of the influencers’ node indicates the total number of retweets and their group, respectively. Nodes that do not bear the color of any influencer, shows the color of the group whose messages the group retweeted the most. The link density represents the number of retweets. Overall, retweets of group B’s posts were dominant. Inside each group were there many retweet interactions, whereas the number of retweets between groups were relatively small. Among each respective group, especially in group B, a tight network was built by the influencers at the hub who frequently retweeted each other’s contents. The network diagram would be uploaded in the following URL. (https://likr.github.io/twitter-analysis2018/)

## Discussion

Scientific communication on SNS (social networking service) has become increasingly important. However, in emergency situations such as a natural disaster where scientific communication is necessary, little is known about how much scientific information is spread and transmitted on Twitter.

Of note, retweets account for roughly half of all the radiation-related tweets and retweets posted within half a year after the Fukushima Daiichi nuclear power plant accident. The majority of the original posts that were retweeted were sent out by accounts defined as “influencers”. In this study, retweets accounted for 49.7% of all tweets and retweets combined. The top 100 accounts received 31.1% of retweets, and the top 200 accounts received 40.0%. Although future research is necessary regarding the extent to which the number of retweets themselves represent the information spreading power of Twitter, our data suggests that it is possible that the majority of information on Twitter is being supplied by very few sources. These findings are comparable to past research results dealing with tweets not related to radiation, such as hate speech targeting foreigners in Japan [34]. Twitter is a social media platform with a high degree of free interaction between individual accounts, but in terms of information spreading, retweets account for half of the total. Influencers can have a stronger impact on information transmitted to the general public rather than the interaction between individuals.

In this study, the majority of influencers (54%) had their personal names attached to their Twitter accounts. News agencies accounted for 15% of influencers, and had a small number of retweets throughout the study period as shown in Fig 3. Some group accounts, which were socially important but not identified by personal names, such as news media and government agencies, did not have a strong influence on information propagation. This result is consistent with a past report showing that Japanese government's tweets were no longer being retweeted once public concerns and doubts have become too strong [35]. These findings suggest that individual accounts bearing real names had more influence on the spread of radiation related information than other accounts. Since there were various opinions on radiation, the general public had trouble ascertaining which information was scientifically correct; and perhaps, judging whether the content of tweet was correct or not, depended on if the sender could be trusted. Scientists should avoid transmitting scientific information in a closed society within their affiliated organizations or open to the public but in an anonymous manner during events that cause social debate such as nuclear accidents and radiation exposure. Although it could give us useful information on how to effectively transmit scientific information, our study has not revealed the mechanism of information spreading by influencers. A further study on their tweets, including those with topics other than radiation, would give us some hint for an effective way of transmitting scientific information to the public.

Interestingly, the ratio of tweets sent by influencers stayed fairly constant since the first month. In the early days after the accident, posts made by group A, B and C were all frequently retweeted; however, the number of retweets received by group A and C showed a rapid decrease, and messages by group B received the majority of retweets one month after the disaster. This tendency remained unchanged over the next six months even after credible scientific information became widely available such as actual measured radiation doses in the environment. We did not investigate why the share of group A and C rapidly reduced, and group B maintained its dominance. We did observe however that group B’s tweets were more emotional than the other groups and involved many criticisms against the government and TEPCO. Such tweets may be easier to propagate widely through SNS than science-based and less emotional information. Scientists should recognize that such emotional exchanges tend to occupy the majority of posts made on SNS. Further research is necessary to understand how to effectively convey scientific but not emotional information through SNS.

The results in this study suggest that retweets were intensively spread within a fixed population, especially within groups with similar document vectors, while intercommunication among groups with different document vectors was small (see Fig 4). These findings are analogous to the fact that at the time of the United States presidential election, each camp accessed only the information its own camp posted on Twitter [36]. While influencers were eventually classified into three groups in the present study, there seems to be a discontinuity in information spreading in group A and B as can be seen in the network diagram (Fig 4). Although this research did not carefully examine the contents of each tweet, the sentiment of tweets concerning safety and danger of radiation is firmly fixed within each group, and the contents of tweets exchanged within each group were clearly differentiated. In group A, information on radiation was transmitted based on relevant scientific evidence, whereas in group B the majority sent out cautionary messages, over-emphasizing or exaggerating the danger of radiation. Therefore, when members of the general public tried to acquire information on radiation, they may have been exposed only to biased information depending on which group of influencers they were following with their Twitter account. Twitter is an interactive social media platform, but information regarding radiological issues was spread mainly through retweeting influencers’ messages; as a result, individuals were found to have received only biased information from a limited number of influencers.

The present study is suggestive when considering how government and international organizations communicate scientific information to the public. As shown in the present study, information on SNS is not limited to only those that are scientifically correct. Contents that are perceived to be more emotional and eye-catching tend be propagated more. A lot of information in Twitter is spread by influencers sharing information with each other. For this reason, the method of unifying the information sources and providing information to the public only from specified organizations is not necessarily optimal as a method of distributing information to the public. Scientists and stakeholders will have to link each other and distribute information in cooperation. In addition, although discussion and public dialogue are important to deepen mutual consent and understanding of controversial issues such as radiation [37, 38], attention must be paid to the possibility that a two-way communication tool like Twitter could be used unilaterally by influencers to spread their own agenda.

## Conclusion

The results of this study showed that retweets account for roughly half of all the tweets related to radiation within half a year after the Fukushima Daiichi nuclear power plant accident. The majority of the retweets were based on original posts sent out by a few hundred accounts defined as “influencers”. The majority of influencers had individual accounts with real names. While the ratio of information spreading by influencers was established and fixed in the first month, retweets were intensively spread within fixed population, especially within groups with similar tweet contents.

## References

[1] Burns TW, O'Connor DJ, Stocklmayer SM. Science communication: a contemporary definition. Public understanding of science. 2003;12(2):183–202.

[2] Nutbeam D. Health literacy as a public health goal: a challenge for contemporary health education and communication strategies into the 21st century. Health Promotion International. 2000;15(3):259–67.

[3] Logan RA. Science mass communication: its conceptual history. Science Communication. 2001;23(2):135–63.

[4] Claussen JE, Cooney PB, Defilippi JM, Fox SG, Glaser SM, Hawkes E, et al Science communication in a digital age: Social media and the American Fisheries Society. Fisheries. 2013;38(8):359–62.

[5] Cann A, Dimitriou K, Hooley T. Social media: A guide for researchers. 2011.

[6] Westerman D, Spence PR, Van Der Heide B. Social media as information source: Recency of updates and credibility of information. Journal of Computer‐Mediated Communication. 2014;19(2):171–83.

[7] Wilcox C. Guest editorial: it’s time to e-volve: taking responsibility for science communication in a digital age Marine Biological Laboratory Woods Hole, Massachusetts; 2012. 10.1086/BBLv222n2p85 22589398

[8] Thomson R, Ito N, Suda H, Lin F, Liu Y, Hayasaka R, et al., editors. Trusting tweets: the Fukushima disaster and information source credibility on Twitter. Proceedings of the 9th International ISCRAM Conference; 2012.

[9] Tanaka Y, Sakamoto Y, Honda H, editors. The impact of posting URLs in disaster-related tweets on rumor spreading behavior. System Sciences (HICSS), Proceedings of 47th Hawaii International Conference on; 2014: IEEE.

[10] Zikmund-Fisher BJ, Dickson M, Witteman HO. Cool but counterproductive: interactive, Web-based risk communications can backfire. J Med Internet Res. 2011;13(3):e60 Epub 2011/08/27. 10.2196/jmir.1665 ; PubMed Central PMCID: PMCPMC3222175. 21868349 PMC3222175

[11] Kwak H, Lee C, Park H, Moon S, editors. What is Twitter, a social network or a news media? Proceedings of the 19th International Conference on World Wide Web; 2010: ACM.

[12] Suh B, Hong L, Pirolli P, Chi EH, editors. Want to be retweeted? Large scale analytics on factors impacting retweet in Twitter network. Social computing (socialcom), 2010 IEEE second international conference on; 2010: IEEE.

[13] Athanasia N, Stavros PT, editors. Twitter as an instrument for crisis response: The Typhoon Haiyan case study. ISCRAM; 2015.

[14] Houston JB, Hawthorne J, Perreault MF, Park EH, Goldstein Hode M, Halliwell MR, et al Social media and disasters: a functional framework for social media use in disaster planning, response, and research. Disasters. 2015;39(1):1–22. 10.1111/disa.12092 25243593

[15] Kogan M, Palen L, Anderson KM, editors. Think local, retweet global: retweeting by the geographically-vulnerable during Hurricane Sandy. Proceedings of the 18th ACM Conference on Computer supported cooperative work & social computing; 2015: ACM.

[16] UNSCEAR. Annex A: Levels and effects of radiation exposure due to the nuclear accident after the 2011 great east-Japan earthquake and tsunami UNSCEAR 2013 report: sources, effects and risks of ionizing radiation. New York: United Nations; 2014.

[17] Normile D. Epidemic of fear. Science. 2016;351(6277):1022–3. 10.1126/science.351.6277.1022 26941301

[18] Murakami M, Sato A, Matsui S, Goto A, Kumagai A, Tsubokura M, et al Communicating with residents about risks following the Fukushima nuclear accident. Asia Pacific Journal of Public Health. 2017;29(2_suppl):74S–89S. 10.1177/1010539516681841 28330403

[19] Goto A, Rudd RE, Lai AY, Yoshida K, Suzuki Y, Halstead DD, et al Leveraging public health nurses for disaster risk communication in Fukushima City: a qualitative analysis of nurses' written records of parenting counseling and peer discussions. BMC Health Services Research. 2014;14(1):129. 24642079 10.1186/1472-6963-14-129 PMC3995308

[20] Kai M. Some lessons on radiological protection learnt from the accident at the Fukushima Dai-ichi nuclear power plant. J Radiol Prot. 2012;32(1):N101–5. Epub 2012/03/08. 10.1088/0952-4746/32/1/N101 . 22394670

[21] Wilensky H, editor. Twitter as a navigator for stranded commuters during the great east Japan earthquake. ISCRAM; 2014.

[22] Toriumi F, Sakaki T, Shinoda K, Kazama K, Kurihara S, Noda I, editors. Information sharing on Twitter during the 2011 catastrophic earthquake. Proceedings of the 22nd International Conference on World Wide Web; 2013: ACM.

[23] Sakaki T, Toriumi F, Shinoda K, Kazama K, Kurihara S, Noda I, et al., editors. Regional analysis of user interactions on social media in times of disaster. Proceedings of the 22nd International Conference on World Wide Web; 2013: ACM.

[24] Ng K-H, Lean M-L. The Fukushima nuclear crisis reemphasizes the need for improved risk communication and better use of social media. Health Physics. 2012;103(3):307–10. 10.1097/HP.0b013e318257cfcb 22850236

[25] Kaigo M. Social media usage during disasters and social capital: Twitter and the Great East Japan earthquake. Keio Communication Review. 2012;34(1):19–35.

[26] Acar A, Muraki Y. Twitter for crisis communication: lessons learned from Japan's tsunami disaster. International Journal of Web Based Communities. 2011;7(3):392–402.

[27] Kudo T, Yamamoto K, Matsumoto Y, editors. Applying Conditional Random Fields to Japanese Morphological Analysis. EMNLP; 2004.

[28] Sato T. Neologism Dictionary based on the Language Resources on the Web for MeCab. 2015.

[29] Rehurek R, Sojka P, editors. Software framework for topic modelling with large corpora In Proceedings of the LREC 2010 Workshop on New Challenges for NLP Frameworks; 2010: Citeseer.

[30] MacQueen J, editor Some methods for classification and analysis of multivariate observations. Proceedings of the fifth Berkeley Symposium on Mathematical Statistics and Probability; 1967: Oakland, CA, USA.

[31] Kodinariya TM, Makwana PR. Review on determining number of Cluster in K-Means Clustering. International Journal. 2013;1(6):90–5.

[32] Hachul S, Jünger M, editors. Drawing large graphs with a potential-field-based multilevel algorithm Graph Drawing; 2004: Springer.

[33] Chimani M, Gutwenger C, Jünger M, Klau GW, Klein K, Mutzel P. The Open Graph Drawing Framework (OGDF). Handbook of Graph Drawing and Visualization. 2013;2011:543–69.

[34] Yamaguchi T. Xenophobia in Action Ultranationalism, Hate Speech, and the Internet in Japan. Radical History Review. 2013;2013(117):98–118.

[35] Li J, Vishwanath A, Rao HR. Retweeting the Fukushima nuclear radiation disaster. Communications of the ACM. 2014;57(1):78–85.

[36] Bessi A, Ferrara E. Social bots distort the 2016 US presidential election online discussion. 2016.

[37] Executive Office of the President Office of Management and Budget. Guidance for Agency Use of Third-Party Websites and Applications. Retrieved June 9, 2018, from https://www.whitehouse.gov/sites/whitehouse.gov/files/omb/memoranda/2010/m10-23.pdf

[38] Public Health England Department for Business, Energy and Industrial Strategy. The Government's Approach to Public Dialogue on Science and Technology. Retrieved June 9, 2018, from https://assets.publishing.service.gov.uk/government/uploads/system/uploads/attachment_data/file/673990/sciencewise-guiding-principles.pdf

